# Polyphenols as the Main Compounds Influencing the Antioxidant Effect of Honey—A Review

**DOI:** 10.3390/ijms251910606

**Published:** 2024-10-01

**Authors:** Aleksandra Wilczyńska, Natalia Żak

**Affiliations:** Department of Quality Management, Gdynia Maritime University, ul. Morska 81-87, 81-225 Gdynia, Poland; n.zak@wznj.umg.edu.pl

**Keywords:** bioactive substances, phenolic compounds, antioxidant activity, pro-health properties

## Abstract

Honey is one of the most valuable components of the human diet. It is considered to be a functional food with health-promoting properties. Honey has bactericidal and bacteriostatic effects; is used to treat wounds and ulcers; relieves stress; supports the treatment of diseases of the digestive and respiratory systems; improves kidney function; and aids in convalescence. The healing and prophylactic effects of honey are closely related to its chemical composition. According to the literature, honey contains over 300 substances belonging to various groups of chemical compounds, some with antioxidant activity, including vitamins and phenolic compounds, mainly flavonoids and phenolic acids. This article provides insight into honey’s chemical composition and its pro-health activities. The antioxidant properties of honey were prioritized.

## 1. Introduction

**Figure 1:**
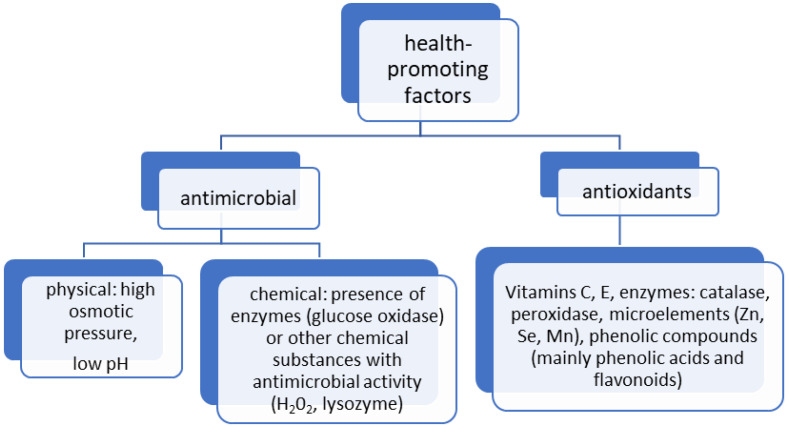
Main factors influencing the health-promoting effects of honey. Source: own study.

Honey, as a natural food product, is one of the most valuable components of the human diet. It is produced by bees, who enrich nectar and honeydew with their own secretions. It is a product of the highest quality, not obtainable by any other means [1]. Honey contains many biologically active substances that regulate digestive processes and heart function, as well as showing, among others, antimicrobial, anti-inflammatory, antidiabetic, antioxidant, and anti-tumoral effects [2]. These properties are closely related to the chemical composition of honey, with both volatile and non-volatile compounds responsible for them, including phenolic compounds, amino acids, enzymes, essential oils, and others [3,4,5,6,7,8,9,10]. However, such a rich and complex chemical composition of honey does not allow it to be considered as a strictly medicinal product. However, in recent years, many articles have appeared in the world literature describing the action of individual honey components at the molecular level. Generally speaking, these components can be divided into those with antimicrobial activity and those with antioxidant activity (Figure 1).

However, since it was discovered that the cause of many diseases (e.g., inflammation, AIDS, hypertension, ischemic disease, neurodegenerative diseases, including Alzheimer’s disease, cancer, stomach ulcers, etc.) may be oxidative stress, researchers have focused on honey’s antioxidant components. Therefore, our goal was to summarize the information about the therapeutic properties of honey antioxidants and the mechanisms of their action.

## 2. Chemical Composition of Honey

As mentioned above, honey’s properties are strictly connected with its chemical composition. The main ingredients of honey are saccharides, dominated by glucose and fructose (85–95%), and sucrose, maltose, and other oligo- and polysaccharides occur in small amounts. Honey also contains a lot of other substances, such as minerals, nitrogen compounds, organic acids, vitamins, minerals, and other phytochemicals. Table 1 summarizes information on the basic chemical composition of honey.

One of the most important effects of honey on human health is its antioxidant effect. Many of the substances included in the honey varieties listed above have antioxidant properties. They include, among others, vitamin C, carotenoids, chlorophyllins, selenium, and some organic acids [20,29]. However, phenolic compounds are mainly responsible for the antioxidant properties of honey. They are represented by flavonoids and phenolic acids. The content of individual flavonoids and phenolic acids depends on the plant source; they are derived from nectar, propolis, and pollen [19,20,35,36,37,38,39].

Phenolic acids are compounds classified as secondary metabolites and are commonly found in plants. There are two subclasses of phenolic acids: hydroxyl derivatives of benzoic or cinnamic acid. Phenolic acids rarely occur as free acids, more often taking the form of esters and glycosides; among others, they form part of the group of hydrolyzing lignins and tannins. Some of the hydroxycinnamic acids are commonly found in ester linkages with carboxylic acids or glucose, while the hydroxybenzoic acids are mostly present as glycosides [40,41,42,43]. The presence of the following phenolic acids has been identified in honey: gallic, ferulic, caffeic, chlorogenic, p-coumaric, syringic, ellagic, and vanillic. Of these acids, the most important in food is gallic acid, a powerful antioxidant; however, other phenolic acids that can be found in honey also have antioxidant as well as antibacterial effects [6,19,20,29,44,45]. They cause, among other effects, cell membrane disruption, bacterial DNA binding, and increased pore formation [29].

**Figure 2:**
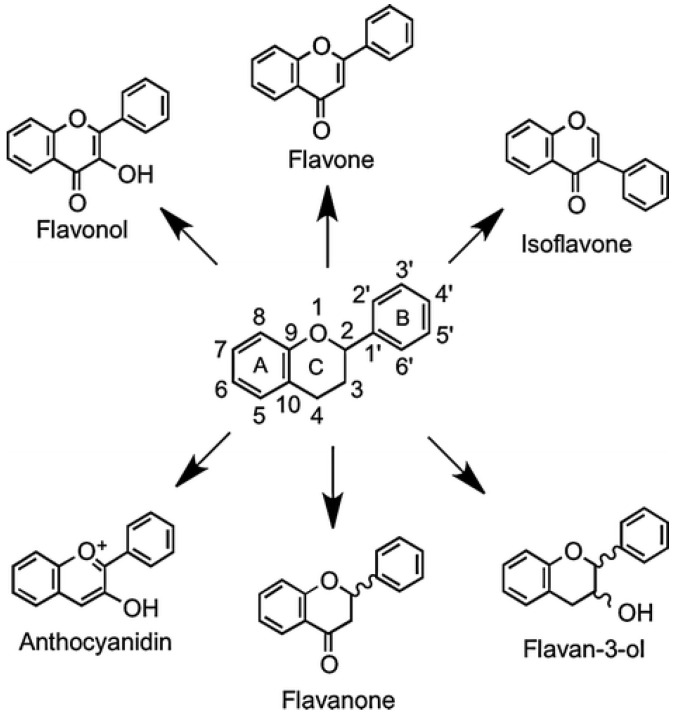
Structure of the flavonoid skeleton [50].

The largest and the most diverse group of honey phenolic compounds are flavonoids. More than 5000 flavonoids have been identified so far. They occur naturally in various plants, where they accumulate mainly in the leaves, flowers, fruits, and seeds. They have also been found in honey and propolis [6,29,44,45,46,47,48]. Flavonoids rarely occur as free molecules, most often appearing in a bound form as glycosides rather than aglycones. Compounds included in this group are similar in terms of their chemical structure. Their distinguishing feature is a diphenylpropane skeleton (C6-C3-C6) with varying degrees of oxidation of the central pyran ring, while most types of flavonoids (except catechins and anthocyanidins) contain a flavone skeleton with a ketone group in position 4. Flavonoids differ in the number and type of substituents (hydroxyl, glycosidic residues, and methoxy groups). The differences between the compounds in individual classes usually result from the different structures of only one terminal ring (Figure 2) [19,39,49]. This affects the properties and biological activity of individual groups of flavonoids.

In honey, the presence of such flavonoids as apigenin, catechin, luteolin, pinocembrin, galangin, and myricetin has been identified [6,29,43,49,50,51,52]. The flavonoid content in honey ranges from a few to several thousand mg/kg of the product and is conditioned by a number of factors, for example, the variety, the climatic conditions during flower nectarization, etc. [38,53,54,55,56,57,58,59,60,61,62].

The table below (Table 2) contains a list of antioxidative components characteristic of honey and their possible pro-health effects.

The above table shows how important the role of honey antioxidants is in preventing many diseases caused by free radicals. It should be emphasized here that the above-described mechanisms are the effects of isolated phytochemical compounds, which have been determined in in vitro studies. It should also be noted that they occur in honey in very small quantities, and, considering the low consumption of honey, they cannot be treated as a significant source of antioxidants in the diet. On the other hand, there is already a lot of evidence supporting the healing effects of honey.

As mentioned above, antioxidant substances present in honey are not only responsible for scavenging free radicals. In particular, polyphenols have a multifaceted effect and are used in the prevention and treatment of many diseases. Based on the existing state of knowledge, it can be concluded that the phenolic compounds contained in honey can strengthen the natural defense mechanisms against oxidative stress and chemical shock [41,85,86,90,91,92,93]. The mechanisms of this impact are very different; honey’s phenolic compounds, among others, participate in the regulation of the expression of genes involved in the reduction in oxidative stress, in the development of amyloid fibrils, and in elevating the expression of the Nrf2 (Nuclear factor erythroid 2-related factor 2) transcription factor, which is responsible for the induction of antioxidant genes [74]. Polyphenols can also stimulate the secretion of antioxidant enzymes such as superoxide dismutase (SOD), catalase (CAT), glutathione peroxidase (GPx), glutathione reductase (GR), and peroxiredoxins [94]. As research has shown, the consumption of honey rich in phenolic compounds increases the antioxidant activity of plasma. It is estimated that replacing at least some of the traditional sugars and confectionery products with honey would result in a significant improvement in defense mechanisms against free radicals [86]. It has also been observed that the antioxidant compounds contained in honey contribute to the protection of pancreatic cells (producers of insulin and glucagon) against oxidative stress and its consequences [87].

The antioxidant properties of flavonoids contained in honey protect the human body against free radicals and create chelating compounds. This helps with the removal of toxic metals (mercury, cadmium, and lead), which can cause damage to the digestive, nervous, and circulatory systems and also influence the development of cancer. For example, the combination of the flavonoid quercetin with lead facilitates the excretion of this metal through urine. Additionally, flavonoids can form chelates with copper, zinc, cadmium, arsenic, nickel, cobalt, and uranium [89].

Polyphenolic compounds in honey are also responsible for their anti-inflammatory effects. Inflammation, along with the presence of oxidative stress, plays a key role in the development and progression of chronic diseases such as cancer, cardiovascular disease, diabetes, arthritis, neurodegenerative diseases, and others. Inflammation and oxidative stress are interconnected. Reactive oxygen species (ROS) produced in mitochondria can cause the production of pro-inflammatory cytokines and mediators, while on the other hand, several cytokines (e.g., TNF-α and IL-1β) can induce the production of ROS from mitochondria. As a result of the correlation between ROS and pro-inflammatory cytokines, metabolic and cellular modifications arise [94]. The aavailable literature shows that the anti-inflammatory effect of honey may be based on various mechanisms. Honey affects the activity of inflammatory mediators in the body—reducing edema formation, inhibiting leukocyte migration, and decreasing the production of ROS and cyclooxygenase-2 activity (COX-2). Honey may also hinder the activation of COX-2 and inducible nitric oxide synthase (iNOS) and reduce prostaglandin synthesis and inflammatory cytokines. Honey polyphenols may also act as agonists of NF-κB receptors and Toll-Like 4 receptors, which are involved in the initiation of inflammation and oxidative stress [95,96,97]. Honey’s polyphenolic compounds can also reduce neuroinflammation brought on by microglia; therefore, they represent a potential solution for lowering neurotoxicity and preventing the evolution of neurological illnesses such as neurodegenerative diseases. This can be achieved by modifying the activity of microglia and reducing the inflammatory response [98].

Honey, due to its anti-inflammatory properties, is used in the treatment of kidney and bladder inflammation and kidney and bladder stones, as well as in the treatment of urinary incontinence in the elderly [85,86]. A few studies have shown that the anti-inflammatory effects of honey can be used in the treatment of inflammatory disorders of the gastrointestinal tract. Among other things, honey supplementation has been studied in terms of its effect on ulcerative colitis. Studies on rats have shown that 1 g/rat of natural honey significantly reduced serum IL-1β and IL-6 levels, while TNF-α, inducible nitric oxide synthase, and caspase-3 were reduced in colonic tissues. Other studies have noted that supplementation with natural Turkish honey for seven days significantly reduced macroscopic and microscopic changes in ulcerative colitis in rats compared to supplementation with synthetic drugs. Similarly, feeding 2.5 g of Manuka honey to rats with chronic gastric ulcers alleviated the disease by promoting the anti-inflammatory cytokine IL-10 and minimizing the pro-inflammatory cytokines TNF-α, IL-1β, and IL-6. Honey has also been used in the treatment of colitis and gastritis. Consumption for seven days reduced ulcerative lesions, microvascular permeability, and gastric myeloperoxidase activity, increasing the efficiency of free radical scavenging [95].

Thanks to the anti-radical and anti-inflammatory effects of polyphenols, honey has also been used as a cardioprotective agent in cardiovascular diseases [99].

Consuming honey lowers LDL cholesterol levels, probably due to the high ability of honey to scavenge free radicals. Through its anti-inflammatory effects, it can positively contribute to the prevention of metabolic and cardiovascular diseases, especially when honey is mixed with other healthy food products [95]. Due to the content of flavonoids, phenolic acids, and vitamin C, honey contributes to improving the efficiency of the heart muscle, regulating blood pressure, and preventing arrhythmia. These compounds cause coronary vasodilation and reduce the risk of thrombus formation. Numerous in vitro and in vivo studies have shown that honey improves the plasma lipid profile, inhibits oxidation, reduces elevated markers of heart damage (CK–MB, AST, ALT), increases the activity of antioxidant enzymes (SOD, GPx, GRx), and increases LDL resistance to oxidation induced by oxidative stress [87,100]. It is assumed that, in this respect, buckwheat honey has the most beneficial effect on the body, and, more specifically, the flavonoid compounds contained in it, i.e., quercetin and rutin. These compounds improve the elasticity and strength of the capillaries, thus protecting them from breaking and limiting the formation of atherosclerotic lesions [90].

The presence of polyphenols in honey also determines their anti-cancer effect. As numerous in vitro studies have shown, honey inhibits the development of cancer cells at various stages of this disease: at initiation, proliferation, and progression. Different mechanisms of anti-tumor activity can be distinguished: the induction of apoptosis, cell cycle arrest, the modulation of oxidative stress, the amelioration of inflammation, the induction of mitochondrial outer membrane permeabilization (MOMP), and the inhibition of angiogenesis. The inhibition of tumor growth has been linked to the antioxidant properties of honey [41].

Polyphenols also have a bactericidal effect against pathogens, both Gram-negative and Gram-positive bacteria. Thanks to the presence of antioxidant compounds and MGO, among others, honey supports the condition of the oral cavity and the treatment of periodontal diseases, halitosis, and paradontosis [91]. Polyphenols also have a positive effect on the development of intestinal microflora. According to various researchers, polyphenols can improve the adhesion and survival of probiotics in the gastrointestinal tract. Polyphenols affect the metabolism and immunity of the intestines and can modulate the composition of the gut microbiota, improving several biochemical markers and risk factors for chronic diseases. There are few supernatural foods that possess a symbiotic trait, which is the combination of a prebiotic carbohydrate with a specific probiotic strain. In this context, prebiotic oligosaccharides and antibacterial compounds found in honey have been developed. For example, Manuka honey has potentially beneficial efficacy in culturing probiotics (*Lactobacillus reuteri*, *Lactobacillus rhamnosus*, and *Bifidobacterium lactis*) and inhibiting other pathogens (*Escherichia coli*, *Salmonella typhimurium*, and *Staphylococcus aureus*) [95]. Honey can promote the growth of some *Lactobacillus* and *Bifidobacterium* bacteria. It has also recently been discovered that encapsulated honey increases the survival of *Bifidobacterium* strains. Also, polyphenol metabolites produced by intestinal microorganisms, including probiotics, exert a number of benefits on the host’s health. These metabolites are often more active than the polyphenols themselves, although the mechanisms of their action have not been identified yet [101,102,103,104,105].

The development of medicine has not been able to fully meet the requirements regarding the effects of a fast lifestyle. Which means that the old methods are being sought or returned to. According to the analysis of the available clinical trial results conducted by Magdas et al. in 2024 [106], eleven main groups can be identified in which honey was confirmed as a therapeutic agent or medicine These include wound care, metabolic disorders, oncology, respiratory diseases, oral health, surgery, gastrointestinal disorders, urogenital disorders, dermatology, sleep disorders, hematology, and nervous system disorders. These studies largely confirmed that researchers around the world are trying to find alternative solutions to chemical drugs derived from natural ingredients. Honey is a very difficult material to use, because the slightest changes resulting from the species of bees, geographical or botanical origin, or the form of sale (directly from the beekeeper or commercially) of honey can lead to various therapeutic effects in the selected range [106]. Summarizing the above-mentioned studies aimed at demonstrating the beneficial effects of honey, especially in the context of metabolic and cardiovascular diseases, it must be stated that they are still insufficient to draw conclusions.

## 3. Methods of Review

The report is based on many years of literature analysis and the authors’ general knowledge. For the purposes of this work, original scientific articles about the therapeutic effects of consuming honey and bee products on the human body were analyzed. The search for the articles was carried out by entering keywords such as honey, bee products, properties of honey, therapeutic effects, natural treatment, and apitherapy. A search of publications from the last 10 years was performed, but some older works were also included. The condition for using older publications was the well-founded knowledge contained in them. Research databases were also searched, such as Google Scholar, Science Direct, MDPI, Knovel, Applied Science & Technology Source, Scopus, Taylor and Francis Online, and EBSCOhost. This study was conducted from June 2023 to May 2024.

## 4. Conclusions

Honey is one of the most valuable products that can be a component of the human diet. Its properties have been known and used for centuries. Honey cannot be considered a medicine, but it is consumed most often for preventive and therapeutic purposes. The healing and prophylactic effects of honey are closely related to the chemical composition of honey, its botanical and geographical origin. A very difficult task in this work was to systematize in a simple way the chemical knowledge about the composition of honey and to reflect it in the mechanisms relating to its impact on the human body. Over the last 20 years, these activities have been undertaken by numerous researchers around the world, and the number of multilingual publications in this aspect may reach 10,000. According to Magdas et al., by 2023, the number of publications in English was 8227 [106].

This paper presents the characteristics of the most important biologically active components of honey and the impacts related to their presence. It can be seen that phenolic compounds are the crucial bioactive components in honey. The protective effect of polyphenolic compounds seems to be particularly important, as they have a very significant role in the prevention and treatment of civilization diseases whose pathogenesis is oxidative stress, i.e., cardiovascular diseases, neurodegenerative diseases, inflammation, hypertension, cancer, ischemic disease, and others.

This article is of a cognitive nature; the authors have based it not only on the world literature, but also on the country they come from. Taking into account research by Stefanis et. al., Poland is one of the 10 countries most frequently publishing research articles on the quality and authenticity of honey [107].

## Figures and Tables

**Table 1:** Basic chemical composition of honey.

Components	Average Content	Comments
Water	17–20%	Some varieties (e.g., heather honey) may contain more—up to 23% [11,12,13,14,15].
Carbohydrates, including:	65–87%	Carbohydrates are the main component of honey. The sugar composition of honey depends on the type of honey, the time of harvesting, and the length of the storage period—unripe honey is characterized by a higher sucrose content. However, the most important factor affecting the content of saccharides is the origin of the honey. The fructose content in honey ranges from 30 to just over 40%. The glucose content is slightly lower and ranges, on average, from 19 to 31%. The ratio of fructose to glucose in most varieties of honey ranges from 1.2 to 1.7. The honeydew honey contain more oligosaccharides and dextrins, while the nectar honey varieties are dominated by mono- and disaccharides [11,12,13,14,15,16,17,18,19].
monosaccharides (glucose, fructose)	67–70%	
disaccharides reducing (maltose, isomaltose, maltulose, isomaltulose, kojibiose, turanose, nigerose, gentiobiose) non-reducing (sucrose, trehalose)	0–15%	
oligosaccharides (reducing and non-reducing)	0.5–10%	
dextrins	1–10%	
Nitrogen compounds, including: amino acids (26 amino acids have been detected in honey, such as proline, alanine, asparagine, glutamine, histidine, glycine, arginine, valine, tyrosine, cysteine, lysine and others).	0.25–3%	Nitrogen compounds appear in honey in small quantities, and these are simple proteins: albumins and globulins, enzymes, and free amino acids. Honey contains 175 mg of free amino acids per 100 g, of which the basic one is proline, constituting about 50–85% of all amino acids. The proline content is a measure of the adulteration of honey with sucrose. Honey enzymes are of both plant origin (from pollen and honeydew) and animal origin (bee glands). They play the role of effective biological catalysts in decomposition and synthesis reactions, e.g., invertase decomposes sucrose, and diastase participates in starch hydrolysis [20,21,22,23,24].
proteins (mainly albumins and globulins)	0.2%	
enzymes (amylases, invertase, catalase, phosphatases, lysozyme and glucose oxidase)	0.2–1%	
Organic acids (gluconic, citric, lactic, malic, succinic, butyric, propionic, tartaric, oxalic, linoleic, linolenic).	0.01–1.5%	Gluconic acid constitutes 70–90% of organic acids in honey. Organic acids determine the taste and the aroma of honey as well as its microbial properties. Just like the content of other components, the acid content is mainly determined by the botanical origin of honey [15,18,25,26].
Inorganic acids (hydrochloric and phosphoric)	0.03–0.1%	
Vitamins (A, C, D, K, B_1_, B_2_, PP, B_6_, B_5_ B_9_, H) [2,15,27,28].	0.04%	
Mineral substances (about 47 elements: potassium, silicon, sodium, iron, copper, magnesium, manganese, sulfur, nickel, phosphorus, chlorine, cobalt, iodine, zinc, palladium, arsenic, strontium, aluminum, tungsten, lead, chromium, titanium, barium, molybdenum, vanadium, tin, silver) [2,10,29,30].	Blossom honey contains 0.05–0.50% of minerals, nectar-honeydew honey—0.32–0.52%, and honeydew honey about 1% of minerals.	The mineral content is influenced by environmental factors, primarily the content of mineral substances in the soil where melliferous plants are grown [24,30,31,32,33].
Aromatic substances (about 200): aldehydes and ketones (formic, acetic and isobutyric aldehydes as well as acetone and diacetyl), polyphenolic compounds, esters, higher aliphatic alcohols. Dyes—carotenoids (carotene and xanthophyll), flavonoids (and the compounds formed by their combination), chlorophyll and anthocyanins, and tannins [29,33,34].	0.07–0.1%	Dyes, ethereal oils, and other aromatic substances in honey are of the floral origin which determines their organoleptic properties. The honey flavor is connected primarily with aldehydes, ketones, esters, higher alcohols, and polyphenolic compounds [19,29,30,33]. The content of aromatic substances may decrease during heating and long storage [18,35].

Own elaboration based on: [2,10,11,12,13,14,15,16,17,18,19,20,21,22,23,24,25,26,27,28,29,30,31,32,33,34].

**Table 2:** Summary of the effects and actions of selected antioxidants from honey.

Component(s)	Mechanisms of Action	Pro-Health Effects	Source
Vitamins C, A, E	Among the antioxidant vitamins, vitamin C is found in the largest amounts in honey. Vitamin C reduces ROS levels and thus prevents the oxidation of lipids, proteins, and DNA, recovers vasodilation, and decreases nitrate tolerance. Vitamin A and β-carotenes improve oxidative stress and cognitive function and reduce toxic amyloid β by inhibiting amyloid β oligomerization and aggregation in a streptozotocin-induced Alzheimer’s disease mouse model. It was also reported that retinoic acid plays a key role in the inhibition of hepatic stellate cell activation (an effector of hepatocellular carcinoma) via suppressing the thioredoxin-interacting protein and reducing oxidative stress levels. Vitamin E reduces the markers of oxidative stress, increases glutathione peroxidase and superoxide dismutase, changes/improves the total antioxidant capacity and glycemic control, and delays the onset as well as the progression of type 2 diabetes. However, considering the content of these vitamins in honey, their contribution to the development of antioxidant and health-promoting properties of honey is insignificant.	Nutritional Properties Antioxidant Effect	[63,64,65,66]
Macro and microelements	Macro- and microelements are compounds necessary to ensure the proper development, growth, and other vital functions of the human body. They regulate a wide array of physiological mechanisms with substantial specificity and selectivity, as components of enzymes and other organic molecular complexes. In honey, trace elements such as copper, zinc, selenium and magnesium, which have antioxidant properties, have been detected. Selenium (Se) is a crucial component of the glutathione peroxidase enzyme, which may eliminate lipid hydroperoxides and hydrogen peroxide (H_2_O_2_). Copper (Cu), as well as zinc (Zn), are necessary building blocks for a number of enzymes, including those involved in oxidation–reduction reaction and Cu–Zn superoxide dismutase (SOD). Manganese (Mn) is an essential co-factor in enzymatic processes connected to the metabolic control of gene expression. Similar to vitamins, the antioxidant effect of honey’s macro- and microelements is of little importance.	Nutritional Properties Antioxidant Properties	[67]
Polyphenols (phenolic acids and flavonoids)	Polyphenols have a protective effect on a number of diseases, e.g., cardiovascular diseases, cancer, neurodegenerative diseases, diabetes, etc. Honey flavonoids exhibit antioxidant activity and also have the ability to inhibit some pro-inflammatory enzymes (cyclooxygenases COX, lipoxygenases LOX, inducible nitric oxide synthase iNOS) and mediators (nitric oxide, cytokines and chemokines). They reduce the oxidation of LDL and lipids and stimulate the maintenance of lipid parameters at the appropriate level, which prevents the clogging of arteries and prevents the effects of atherosclerotic changes in the blood vessels. Flavonoids counteract tumor development by inhibiting tumor-producing enzymes, blocking the activity of certain hormones, and interfering with the delivery of oxygen and other components necessary for tumor formation. These compounds are responsible, among other things, for reducing oxidative stress, decreasing apoptosis, necrosis, brain atrophy and behavioral and neurological deficits. Recently, there have been reports on the use of honey’s phenolic compounds for the treatment of Alzheimer’s disease. They influence, among other things, neurodegeneration associated with amyloid pathology and ischemia in proteinopathy. Honey polyphenols can also prevent numerous neurodegenerative diseases by protecting neurons from oxidative damage, enhancing neuronal function and regeneration, protecting neurons from Ab-induced neuronal injury and neurotoxicity, protecting hippocampal cells against nitric oxide-induced toxicity, and modulating neuronal and glial cell signaling pathways. For example, ferulic acid exerted neuroprotective effects in a mouse model of Parkinson’s disease by decreasing the levels of phospho-Akt, phospho-pyruvate dehydrogenase kinase-1, and phospho-Bad and increasing the caspase-3 levels. The mechanisms of action of particular polyphenols are different and depend mainly on the molecule structure, e.g., kaempferol, luteolin, chrysin, pinocembrin, and gallic acid induce apoptosis, while apigenin promotes interleukin 1b and tumor necrosis factor. Polyphenols also neutralize oxidative stress in various ways. Caffeic acid inhibits oxidative stress in iron-overloaded rats by reducing lipid peroxidation and increasing vitamin E levels in the plasma. Quercetin reduces oxidative stress by scavenging free radicals, chelating metal ions, and inhibiting xanthine oxidase and lipid peroxidation. Kaempferol reduces oxidative stress caused by glutamate in the mouse hippocampal cell line HT-22 by blocking ROS generation, and also blocks oxidative stress in granule cells during low-potassium-induced apoptosis. Through antioxidant pathways, honey’s polyphenols ameliorate cholesterol and cardiac enzyme levels. Polyphenols also exhibit bactericidal effects against both Gram-negative and Gram-positive bacteria. Their antibacterial mechanism is based on inhibiting bacterial biofilm formation or inactivating enzymes. In alkaline conditions (pH 7.0–8.0), polyphenols can display pro-oxidative properties, inhibiting microbial growth by accelerating hydroxyl radical formation and oxidative strand breakage in DNA. They could also support the production of considerable amounts of H_2_O_2_ via a non-enzymatic pathway.	Anti-Allergic Effect Antioxidant, Anti-Inflammatory, and Anty-hiperlipidemic Properties Application in Disorders of the Immune System Cardio- and NeuroProtective Effects Antibacterial and Antiviral Effect	[29,41,46,47,68,69,70,71,72,73,74,75,76,77,78,79,80,81,82,83,84,85,86]
Enzymes (e.g., catalase, peroxidase)	They enable digestion by breaking down complex molecules such as sugars, fats, and carbohydrates into simpler elements. The enzyme glucose oxidase, most likely originating from the digestive tract of bees, is responsible for the bacteriostatic and bactericidal properties of honey. This enzyme belongs to the group of oxidoreductases and catalyzes the oxidation of glucose to glucono-δ-lactone. The by-product of this reaction is H_2_O_2_. Catalase is also included in the group of oxidoreductases. This enzyme comes from nectar, honeydew, or pollen. Catalase acts as a regulator of the H_2_O_2_ content, catalyzing the reaction of its decomposition into water and molecular oxygen, thus contributing to the reduction in the bactericidal and bacteriostatic properties of honey.	Antioxidant Effect	[87,88,89]

Source: Own elaboration based on: [41,63,64,65,66,67,68,69,70,71,72,73,74,75,76,77,78,79,80,81,82,83,84,85,86,87,88,89].

## References

[1] (2014). DIRECTIVE2014/63/EU of the European Parliament and of the Council of 15 May 2014 Amending Council Directive 2001/110/EC Relating to Honey, Official Journal of the European Union L 164/1.

[2] Laaroussi H., Bouddine T., Bakour M., Ousaaid D., Lyoussi B. (2020). Physicochemical Properties, Mineral Content, Antioxidant Activities, and Microbiological Quality of *Bupleurum spinosum* Gouan Honey from the Middle Atlas in Morocco. J. Food Qual..

[3] Bobis O., Moise A.R., Ballesteros I., Reyes E.S., Durán S.S., Sánchez-Sánchez J., Cruz-Quintana S., Giampieri F., Battino M., Alvarez-Suarez J.M. (2020). Eucalyptus Honey: Quality Parameters, Chemical Composition and Health-Promoting Properties. Food Chem..

[4] Bogdanov S., Jurendic T., Sieber R., Gallmann P. (2013). Honey for Nutrition and Health: A Review. J. Apic. Res..

[5] Boussaid A., Chouaibi M., Rezig L., Hellal R., Donsì F., Ferrari G., Hamdi S. (2018). Physicochemical and Bioactive Properties of Six Honey Samples from Various Floral Origins from Tunisia. Arab. J. Chem..

[6] Cheung Y., Meenu M., Yu X., Xu B. (2019). Phenolic Acids and Flavonoids Profiles of Commercial Honey from Different Floral Sources and Geographic Sources. Int. J. Food Prop..

[7] Erban T., Shcherbachenko E., Talacko P., Harant K. (2019). The Unique Protein Composition of Honey Revealed by Comprehensive Proteomic Analysis: Allergens, Venom-like Proteins, Antibacterial Properties, Royal Jelly Proteins, Serine Proteases, and Their Inhibitors. J. Nat. Prod..

[8] Marić A., Jovanov P., Sakać M., Novaković A., Hadnadev M., Pezo L., Mandić A., Milićewić N., Durović A., Gadžurić S. (2021). A comprehensive study of parameters correlated with honey health benefits. RSC Adv..

[9] Pauliuc D., Ciursă P., Ropciuc S., Dranca F., Oroian M. (2021). Physicochemical Parameters Prediction and Authentication of Different Monofloral Honeys Based on FTIR Spectra. J. Food Compos. Anal..

[10] Sakać M.B., Jovanov P.T., Marić A.Z., Pezo L.L., Kevrešan Ž.S., Novaković A.R., Nedeljković N.M. (2019). Physicochemical Properties and Mineral Content of Honey Samples from Vojvodina (Republic of Serbia). Food Chem..

[11] Oddo L.P., Piro R., Bruneau É., Guyot-Declerck C., Ivanov T., Piskulová J., Flamini C., Lheritier J., Morlot M., Russmann H. (2004). Main European unifloral honeys: Descriptive sheets. Apidologie.

[12] Bogdanov S. (2009). Physical Properties of Honey. Book of Honey; Chapter 4 Bee Product Science. www.bee-hexagon.net.

[13] Crăciun M.E., Pârvulescu O.C., Donise A.C., Dobre T., Stanciu D.R. (2020). Characterization and classification of Romanian acacia honey based on its physicochemical parameters and chemometrics. Sci. Rep..

[14] Manzanares A.B., García Z.H., Galdón B.R., Rodríguez E.R., Romero C.D. (2014). Physicochemical characteristics of minor monofloral honeys from Tenerife, Spain. LWT—Food Sci. Technol..

[15] Gündoğdu E., Çakmakç S., Şat İ.G. (2019). An Overview of Honey: Its Composition, Nutritional and Functional Properties. J. Food Sci. Eng..

[16] El Sohaimy S.A., Masry S.H.D., Shehata M.G. (2015). Physicochemical characteristics of honey from different origins. Ann. Agric. Sci..

[17] Iglesias M.T., Martian-Alvarez P.J., Polo M.C., Lorenzo C., Gonzalez M., Pueyo E.N. (2006). Changes in the free amino acid contents of honeys during storage at ambient temperature. J. Agric. Food Chem..

[18] Rybak-Chmielewska H. (2007). Changes in the carbohydrate composition of honey undergoing during storage. J. Apic. Sci..

[19] Bonté F., Desmoulière A. (2013). Le miel: Origine et composition. Actual. Pharm..

[20] Janiszewska K., Aniołowska M., Nowakowski P. (2012). Free amino acids content of honeys from Poland. Pol. J. Food Nutr. Sci..

[21] Hermosi’n I., Chicón R.M., Dolores Cabezudo M. (2002). Free amino acid composition and botanical origin of honey. Food Chem..

[22] Pita-Calvo C., Vázquez M. (2017). Differences between honeydew and blossom honeys: A review. Trends Food Sci. Technol..

[23] Won S., Lee D., Ko S.H., Kim J., Rhee H. (2008). Honey major protein characterization and its application to adulteration detection. Food Res. Int..

[24] Sak-Bosnar M., Sakac N. (2012). Direct potentiometric determination of diastase activity in honey. Food Chem..

[25] Villacrés-Granda I., Coello D., Proaño A., Ballesteros I., Roubik D.W., Jijón G., Granda-Albuja G., Granda-Albuja S., Abreu-Naranjo R., Maza F. (2021). Honey quality parameters, chemical composition and antimicrobial activity in twelve Ecuadorian stingless bees (Apidae: Apinae: Meliponini) tested against multiresistant human pathogens. LWT—Food Sci. Technol..

[26] Won S.A., Li C., Kim J., Rhee H. (2009). Immunological characterization of honey major protein and its application. Food Chem..

[27] Da Silva P.M., Gauche C., Gonzaga L.V., Oliveira Costa A.C., Fett R. (2016). Honey: Chemical composition, stability and authenticity. Food Chem..

[28] Wang J., Li Q.X., Taylor S.L. (2011). Chemical Composition, Characterization, and Differentiation of Honey Botanical and Geographical Origins. Advances in Food Nutrition Research.

[29] Madejczyk M., Baralkiewicz D. (2008). Characterization of Polish rape and honeydew honey according to their mineral contents using ICP-MS and F-AAS/AES. Anal. Chim. Acta.

[30] Alqarni A.S., Owayss A.A., Mahmoud A.A. (2012). Mineral content and physical properties of local and imported honeys in Saudi Arabia. J. Saudi Chem. Soc..

[31] Moreira R.F.A., Maria C.A.B., Pietroluongo M., Trugo L.C. (2007). Chemical changes in the non-volatile fraction of Brazilian honeys during storage under tropical conditions. Food Chem..

[32] Zammit Young G.-W., Blundell R. (2023). A review on the phytochemical composition and health applications of honey. Heliyon.

[33] Karabagias I.K., Badeka A., Kontakos S., Karabournioti S., Kontominas M.G. (2014). Characterisation and classification of Greek pine honeys according to their geographical origin based on volatiles, physicochemical parameters and chemometrics. Food Chem..

[34] Bertoncelj J., Doberšek U., Jamnik M., Golob T. (2007). Evaluation of the phenolic content, antioxidant activity and colour of Slovenian honey. Food Chem..

[35] Mato I.S., Huidobro J.F., Simal-Lozano J.S., Sancho M.T. (2006). Rapid determination of nonaromatic organic acids in honey by capillary zone electrophoresis with direct ultraviolet detection. J. Agric. Food Chem..

[36] Miguel M., Antunes M., Faleiro M. (2017). Honey as a Complementary Medicine. Integr. Med. Insights.

[37] Combarros-Fuertes P., Estevinho L.M., Dias L.G., Castro J.M., Tomás-Barberán F.A., Tornadijo M.E., Fresno-Baro J.M. (2019). Bioactive Components and Antioxidant and Antibacterial Activities of Different Varieties of Honey: A Screening Prior to Clinical Application. J. Agric. Food Chem..

[38] Bucekova M., Jardekova L., Juricova V., Bugarova V., Di Marco G., Gismondi A., Leonardi D., Farkasovska J., Godocikova J., Laho M. (2019). Antibacterial Activity of Different Blossom Honeys: New Findings. Molecules.

[39] Pyrzynska K., Biesaga M. (2009). Analysis of phenolic acids and flavonoids in honey. Trends Anal. Chem..

[40] Kędzierska-Matysek M., Stryjecka M., Teter A., Skałecki P., Domaradzki P., Florek M. (2021). Relationships between the Content of Phenolic Compounds and the Antioxidant Activity of Polish Honey Varieties as a Tool for Botanical Discrimination. Molecules.

[41] Cianciosi D., Forbes-Hernández T.Y., Afrin S., Gasparrini M., Reboredo-Rodriguez P., Manna P.P., Zhang J., Bravo Lamas L., Martínez Flórez S., Agudo Toyos P. (2018). Phenolic Compounds in Honey and Their Associated Health Benefits: A Review. Molecules.

[42] Hernanz Vila M.D., Jara Palacios M.J., Santos Morcillo J.L., Gómez Pajuelo A., Heredia Mira F.J., Terrab Benjelloun A. (2023). The profile of phenolic compounds by HPLC-MS in Spanish oak (Quercus) honeydew honey and their relationships with color and antioxidant activity. LWT—Food Sci. Technol..

[43] Borges A., Ferreira C., Saavedra M.J., Simões M. (2013). Antibacterial activity and mode of action of ferulic and gallic acids against pathogenic bacteria. Microb. Drug Resist..

[44] Działo M., Mierziak J., Korzun U., Preisner M., Szopa J., Kulma A. (2016). The potential of plant phenolics in prevention and therapy of skin disorders. Int. J. Mol. Sci..

[45] Khan F., Bamunuarachchi N.I., Tabassum N., Kim Y.-M. (2021). Caffeic acid and its derivatives: Antimicrobial drugs toward microbial pathogens. J. Agric. Food Chem..

[46] Ranneh Y., Faisal Ali F., Zarei M., Akim A.M., Hamid H.A., Khazaai H. (2018). Malaysian stingless bee and Tualang honeys: A comparative characterization of total antioxidant capacity and phenolic profile using liquid chromatography-mass spectrometry. LWT—Food Sci. Technol..

[47] Górniak I., Bartoszewski R., Króliczewski J. (2018). Comprehensive review of antimicrobial activities of plant flavonoids. Phytochem. Rev. Proc. Phytochem. Soc. Eur..

[48] Procházková D., Boušová I., Wilhelmová N. (2011). Antioxidant and prooxidant properties of flavonoids. Fitoterapia.

[49] Cushnie T., Lamb A.J. (2005). Antimicrobial activity of flavonoids. Int. J. Antimicrob. Agents.

[50] Del Rio D., Rodriguez-Mateos A., Spencer J.P., Tognolini M., Borges G., Crozier A. (2013). Dietary (poly)phenolics in human health: Structures, bioavailability, and evidence of protective effects against chronic diseases. Antioxid. Redox Signal..

[51] Nešović M., Gašić U., Tosti T., Horvacki N., Šikoparija B., Nedić N., Blagojević S., Ignjatović L., Tešić Ž. (2020). Polyphenol profile of buckwheat honey, nectar and pollen. R. Soc. Open Sci..

[52] Tanleque-Alberto F., Juan-Borrás M., Escriche I. (2020). Antioxidant characteristics of honey from Mozambique based on specific flavonoids and phenolic acid compounds. J. Food Compos. Anal..

[53] Yayinie M., Atlabachew M., Tesfaye A., Hilluf W., Reta C., Alemneh T. (2022). Polyphenols, flavonoids, and antioxidant content of honey coupled with chemometric method: Geographical origin classification from Amhara region, Ethiopia. Int. J. Food Prop..

[54] Lv C., Yang J., Liu R., Lu Q., Ding Y., Zhang J., Deng J. (2018). A comparative study on the adsorption and desorption characteristics of flavonoids from honey by six resins. Food Chem..

[55] Gašić U., Kečkeš S., Dabić D., Trifković J., Milojković-Opsenica D., Natić M., Tešić Ž. (2014). Phenolic profile and antioxidant activity of Serbian polyfloral honeys. Food Chem..

[56] Iurlina M.O., Saiz A.I., Fritz R., Manrique G.D. (2009). Major flavonoids of Argentinean honeys. Optimisation of the extraction method and analysis of their content in relationship to the geographical source of honeys. Food Chem..

[57] Bertoncelj J., Polak T., Kropf U., Korošec M., Golob T. (2011). LC-DAD-ESI/MS analysis of flavonoids and abscisic acid with chemometric approach for the classification of Slovenian honey. Food Chem..

[58] Formosa J.P. (2017). Chemical Profiling of Honey Produced in the Maltese Islands. Master’s Thesis.

[59] Griep M.A., Blood S., Larson M.A., Koepsell S.A., Hinrichs S.H. (2007). Myricetin inhibits *Escherichia coli* DnaB helicase but not primase. Bioorg. Med. Chem..

[60] Escriche I., Kadar M., Juan-Borras M., Domenech E. (2011). Using flavonoids compounds and headspace voiltale profile for botanical authentication of lemon and orange honeys. Food Res. Int..

[61] Estevinho L., Pereira A.P., Moreira L., Dias L.G., Pereira E. (2008). Antioxidant and antimirobial effects of phenolic compounds extracts of Northeast Portugal honey. Food Chem. Toxicol..

[62] Kenjeric D., Mandic M.L., Primorac L., Bubalo D., Perl A. (2007). Flavonoid profile of Robinia honeys produced in Croatia. Food Chem..

[63] Fatima M.T., Bhat A.A., Nisar S., Fakhro K.A., Akil A.S.A.-S. (2023). The role of dietary antioxidants in type 2 diabetes and neurodegenerative disorders: An assessment of the benefit profile. Heliyon.

[64] Padayatty S.J., Katz A., Wang Y., Eck P., Kwon O., Lee J.-H., Chen S., Corpe C., Dutta A., Dutta S.K. (2003). Vitamin C as an Antioxidant: Evaluation of Its Role in Disease Prevention. J. Am. Coll. Nutr..

[65] Gęgotek A., Skrzydlewska E. (2022). Antioxidative and Anti-Inflammatory Activity of Ascorbic Acid. Antioxidants.

[66] Shimizu H., Tsubota T., Kanki K., Shiota G. (2018). All-trans retinoic acid ameliorates hepatic stellate cell activation via suppression of thioredoxin interacting protein expression. J. Cell. Physiol..

[67] El-Sayed A., Ebissy E., Mohamed R., Ateya A. (2024). Effects of antioxidant vitamins (A, D, E) and trace elements (Cu, Mn, Se, Zn) administration on gene expression, metabolic, antioxidants and immunological profiles during transition period in dromedary camels. BMC Vet. Res..

[68] Velimirović D., Tošić S., Mitić S., Pavlović A., Rašić Mišić I., Stojanović G. (2023). Mineral, phenolic content and antioxidant activity of selected honey samples consumed in Serbia. J. Apic. Res..

[69] Kenjeric D., Mandic M.L., Primorac L., Cacic F.A. (2008). Flavonoid pattern of sage (*Salvia officinalis* L.) unifloral honeys. Food Chem..

[70] Kim S., Woo E.-R., Lee D.G. (2020). Apigenin promotes antibacterial activity via regulation of nitric oxide and superoxide anion production. J. Basic Microbiol..

[71] Kassim M., Achoui M., Mustafa M.R., Mohd M.A., Yusoff K.M. (2010). Ellagic acid, phenolic acids and flavonoids in Malaysian honey extracts demonstrate in vitro antiflammatory activity. Nutr. Res..

[72] Guo Y., Liu Y., Zhang Z., Chen M., Zhang D., Tian C., Liu M., Jiang G. (2020). The antibacterial activity and mechanism of action of Luteolin against *Trueperella pyogenes*. Infect. Drug Resist..

[73] Silva B., Biluca F.C., Gonzaga L.V., Fett R., Monguilhott Dalmarco E., Caon T., Oliveira Costa A.C. (2021). In vitro anti-inflammatory properties of honey flavonoids: A review. Food Res. Int..

[74] Candiracci M., Citterio B., Piatti E. (2012). Antifungal activity of the honey flavonoid extract against *Candida albicans*. Food Chem..

[75] Soobrattee M.A., Neergheen V.S., Luximon-Ramma A., Arouma O.I., Bahorun T. (2005). Phenolics as potential antioxidant therapeutic agents: Mechanism and actions. Mutat. Res..

[76] Pluta R., Miziak B., Czuczwar S.J. (2023). Apitherapy in Post-Ischemic Brain Neurodegeneration of Alzheimer’s Disease Proteinopathy: Focus on Honey and Its Flavonoids and Phenolic Acids. Molecules.

[77] Tichonow A.I., Bondarenko L.A., Jarnych T.G., Szpyczak O.S., Kowal W.M., Skrypnik-Tichonow R.I. (2017). Miód Naturalny w Medycynie i Farmacji (Pochodzenie, Właściwości, Zastosowanie, Preparaty Lecznicze).

[78] Rosiak E., Jaworska D. (2019). Probiotic and prebiotic properties of bee honey in terms of their quality and health safety. Żywn. Nauka Technol. Jakość.

[79] Hossen M.S., Ali M.Y., Jahurul M.H.A., Abdel-Daim M.M., Gan S.H., Khalil M.I. (2017). Beneficial roles of honey polyphenols against some human degenerative diseases: A review. Pharmacol. Rep..

[80] Bueno-Costa F.M., Zambiazi R.C., Bohmer B.W., Chaves F.C., da Silva W.P., Zanusso J.T., Dutra I. (2016). Antibacterial and antioxidant activity of honeys from the state of Rio Grande do Sul, Brazil. LWT—Food Sci. Technol..

[81] Terzo S., Mulè F., Amato A. (2020). Honey and obesity-related dysfunctions: A summary on health benefits. J. Nutr. Biochem..

[82] Lori G., Cecchi L., Mulinacci N., Melani F., Caselli A., Cirri P., Paoli P. (2019). Honey extracts inhibit PTP1B, upregulate insulin receptor expression, and enhance glucose uptake in human HepG2 cells. Biomed. Pharmacother..

[83] Manna P., Jain S.K. (2015). Obesity, oxidative stress, adipose tissue dysfunction, and the associated health risks: Causes and therapeutic strategies. Metab. Syndr. Relat. Disord..

[84] Bobiş O., Dezmirean D.S., Moise A.R. (2018). Honey and diabetes: The importance of natural simple sugars in diet for preventing and treating different type of diabetes. Oxid. Med. Cell. Longev..

[85] Meo S.A., Al-Asiri S.A., Mahesar A.L., Ansari M.J. (2017). Role of honey in modern medicine. Saudi J. Biol. Sci..

[86] Schramm D.D., Karim M., Schrader H.R., Holt R.R., Cardetti R., Keen C.L. (2003). Honey with high levels of antioxidants can provide protection to healthy human subjects. J. Agric. Food. Chem..

[87] Ahmed S., Sulaiman S.A., Baig A.A., Ibrahim M., Liaqat S., Fatima S., Othman N.H. (2018). Honey as a potential natural antioxidant medicine: An insight into its molecular mechanisms of action. Oxid. Med. Cell. Longev..

[88] Soares S., Amaral J.S., Oliveira M.B.P.P., Mafra I. (2017). A Comprehensive review on the main honey authentication issues: Production and origin. Compr. Rev. Food Sci. Food Saf..

[89] Samarghandian S., Farkhondeh T., Samini F. (2017). Honey and Health: A Review of Recent Clinical Research. Pharmacogn. Res..

[90] Ramsay E.I., Rao S., Madathil L., Hegde S.K., Baliga-Rao M.P., George T., Baliga M.S. (2019). Honey in oral health and care: A mini review. J. Oral Biosci..

[91] Kaur S., Mirza A., Singh J. (2017). Recent advances of honey in modern medicines: A review. J. Pharmacogn. Phytochem..

[92] Dżugan M., Tomczyk M., Sowa P., Grabek-Lejko D. (2018). Antioxidant activity as biomarker of honey variety. Molecules.

[93] Küçük M., Kolayli S., Karaoğlu S., Ulusoy E., Baltaci C., Candan F. (2007). Biological activities and chemical composition of three honeys of different types from Anatolia. Food Chem..

[94] Becerril-Sánchez A.L., Quintero-Salazar B., Dublán-García O., Escalona-Buendía H.B. (2021). Phenolic Compounds in Honey and Their Relationship with Antioxidant Activity, Botanical Origin, and Color. Antioxidants.

[95] Ranneh Y., Akim A.M., Hamid H.A., Khazaai H., Fadel A., Zakaria Z.A., Albujja M., Bakar M.F.A. (2021). Honey and Its Nutritional and Anti-Inflammatory Value. BMC Complement. Med. Ther..

[96] Chua L.S., Ismail N.I.M. (2015). Anti-inflammatory and anti-microbial activities of selected honey samples. Asian J. Agric. Res..

[97] Cho H., Yun C.-W., Park W.-K., Kong J.-Y., Kim K.S., Park Y., Lee S., Kim B.-K. (2004). Modulation of the activity of pro-inflammatory enzymes, COX-2 and iNOS, by chrysin derivatives. Pharmacol. Res..

[98] Riaz T., Akram M., Laila U., Khalil M.T., Zainab R., Iftikhar M., Ozdemir F.A., Sołowski G., Alinia-Ahandani E., Altable M. (2023). A review of pharmacology and medicinal properties of honey. IAIM.

[99] Bt Hj Idrus R., Sainik N.Q.A.V., Nordin A., Saim A.B., Sulaiman N. (2020). Cardioprotective Effects of Honey and Its Constituent: An Evidence-Based Review of Laboratory Studies and Clinical Trials. Int. J. Environ. Res. Public Health.

[100] Mohan A., Quek S.-Y., Gutierrez-Maddox N., Gao Y., Shu Q. (2017). Effect of Honey in Improving the Gut Microbial Balance. Food Qual. Saf..

[101] Favarin L., Laureano-Melo R., Luchese R.H. (2015). Survival of free and microencapsulated Bifidobacterium: Effect of honey addition. J. Microencapsul..

[102] Catalkaya G., Venema K., Lucini L., Rocchetti G., Delmas D., Daglia M., De Filippis A., Xiao H., Quiles J.L., Xiao J. (2020). Interaction of Dietary Polyphenols and Gut Microbiota: Microbial Metabolism of Polyphenols, Influence on the Gut Microbiota, and Implications on Host Health. Food Front..

[103] Ray S.K., Mukherjee S. (2021). Evolving Interplay Between Dietary Polyphenols and Gut Microbiota—An Emerging Importance in Healthcare. Front. Nutr..

[104] de Souza E.L., de Albuquerque T.M.R., Dos Santos A.S., Massa N.M.L., de Brito Alves J.L. (2019). Potential Interactions among Phenolic Compounds and Probiotics for Mutual Boosting of Their Health-Promoting Properties and Food Functionalities—A Review. Crit. Rev. Food Sci. Nutr..

[105] Jones R. (2009). Honey and healing through the ages. J. ApiProduct ApiMedical Sci..

[106] Magdas T.M., David M., Hategan A.R., Filip G.A., Magdas D.A. (2024). Geographical Origin Authentication—A Mandatory Step in the Efficient Involvement of Honey in Medical Treatment. Foods.

[107] Stefanis C., Stavropoulou E., Giorgi E., Voidarou C., Constantinidis Vrioni G., Tsakris A. (2023). Honey’s antioxidant and antimicrobial properties: A bibliometric study. Antioxidants.

